# Morphological Seed Characterization of Common (*Phaseolus vulgaris* L.) and Runner (*Phaseolus coccineus* L.) Bean Germplasm: A Slovenian Gene Bank Example

**DOI:** 10.1155/2019/6376948

**Published:** 2019-01-16

**Authors:** Lovro Sinkovič, Barbara Pipan, Eva Sinkovič, Vladimir Meglič

**Affiliations:** ^1^Crop Science Department, Agricultural Institute of Slovenia, Hacquetova Ulica 17, 1000 Ljubljana, Slovenia; ^2^Department of Agronomy, Biotechnical Faculty, University of Ljubljana, Jamnikarjeva 101, 1000 Ljubljana, Slovenia

## Abstract

Genetic resources comprised of 953 accessions of common (*Phaseolus vulgaris* L.) and 47 accessions of runner (*Phaseolus coccineus* L.) bean from the national Slovene gene bank were characterized using fourteen morphological seed descriptors. Seeds of each accession were evaluated for six quantitative characteristics: seed length, seed thickness, seed width, seed length/width ratio, seed width/thickness ratio, and 100 or 10 seed weight. Furthermore, seeds were evaluated using eight qualitative characteristics: seed colour; number of seed colours; primary/main seed colour; predominant secondary seed colour; distribution of secondary seed colour; seed veining; seed shape; and seed colour (primary and secondary) and coat pattern. For each, common, and runner bean collection, first four components within principal component analysis explained 75.03% and 80.16% of morphological variability, respectively. Regarding Ward's method and squared Euclidian distance, three clusters with the most distinct characteristics were established for each species. The results of morphological seed characterization indicate the origin (Andean, Mesoamerican, putative hybrids between gene pools) and domestication pathways of common and runner bean. This is the first study describing morphological seed characteristics of the entire common and runner bean germplasm conserved in one of the Central European bean collections. The results obtained in this study are serving as the useful information on genetic diversity of common and runner bean accessions at the Slovene gene bank, which could be used for development of new bean varieties for studied seed characteristics.

## 1. Introduction

Genetic diversity or variation between different populations belonging to the same genus resulted from the evolution of crops through the history, in response to different environments and husbandry practices [1].* Phaseolus* spp. beans are valued grain legumes or pulse crops of worldwide importance in terms of human and animal consumption [2–4]. Common bean (*Phaseolus vulgaris* L.) is the most important* Phaseolus* spp. worldwide, while the runner bean (*Phaseolus coccineus* L.) is the third, right after lima bean (*Phaseolus lunatus *L.) [5, 6]. Cultivated common and runner beans were domesticated independently within two centres of diversity, giving rise to two gene pools, i.e., Mesoamerican and Andean [4, 7]. The lack of characterization of the* Phaseolus coccineus* L. germplasm restricts its utilization as donor species for interspecific hybridization and consequently limits its use in other* Phaseolus* spp. breeding programs, i.e., common bean [6].

Ensuring the preservation of future sources is a big challenge for plant geneticists and breeders. Seed gene banks are intended to enable the conservation of the world's crop genetic diversity against the genetic erosion of crops as an unintended consequence of the global uptake of new high-yielding green revolution agricultural varieties [8]. Seed gene banks are facilities dedicated to the medium-term storage, i.e., for a few decades in storage at 5–10°C, or long-term storage, i.e., for many decades in storage at −18 ± 3°C, of samples of seeds as a means of conserving crop or species diversity. A seed gene bank conserving crop varieties is often called a gene bank as the seed samples, i.e., accessions, are used as a source of genes conferring desirable characteristics [9].

Genetic diversity of common bean from Central Europe was studied at the Agricultural Institute of Slovenia by AFLP and microsatellite markers [10–12]. The surveys revealed that extensive diversity resides in common bean cultivated in this area and includes variation beyond the two gene pools, i.e., Andean and Mesoamerican. As revealed by the analysis of a large set of European common bean landraces using chloroplast microsatellites (cpSSRs) and two unlinked nuclear loci (phaseolin;* PvSHP1*) a relatively high proportion of the European bean germplasm might have derived from hybridization between the two gene pools [13, 14]. Recently, 300 Croatian common bean landraces have been evaluated by phaseolin and microsatellite markers. The majority of the studied landraces belong to Andean gene pool, similarly as was found for Slovenian landraces [10]. Genetic diversity of runner bean has been less extensively investigated. The largest set of European landraces, more than 300, was evaluated by cpSSRs and a smaller set was studied also for phenotypic traits [15].

Two main gene pools, Mesoamerican and Andean associated with these two geographical areas, have been described in wild and cultivated common beans. As a result of the domestication process, a great number of varieties showing differences in morpho-agronomical quantitative traits including seed size, seed quality, and plant growing period, were obtained, and this variation has been extensively used in breeding programs or diversity studies [16]. Large or medium seed morphology characteristic was reported for Andean and mostly small seeds for Mesoamerican group genotypes [17]. Purple seed colour was found to be exclusively Andean, while pink, brown, and black predominantly Mesoamerican pool origin. Cream, yellow, and red seed colours were found in both gene pool groups. Andean beans having a tendency for higher iron seed concentration and lower seed zinc concentration than Mesoamerican and putative hybrids between gene pools [18].

In Slovenia, the majority of the bean production is based on local populations and varieties grown by small-scale farmers in low input production systems. Populations are well adapted to the specific growing conditions and microclimate agroenvironments and show a great seed morphological diversity. The aim of the present study was to evaluate the common and runner bean germplasm collections from Slovene gene bank using seed characteristics and to establish clusters of the most distinct genetic resources, based on morphological seed evaluation. Common and runner bean information based on seed descriptors would be the first step for the assessment, description and classification of large bean germplasm collections, to enable better and faster use in future research and development of new varieties.

## 2. Materials and Methods

### 2.1. Plant Material

The plant material used in the present study comprised a total of 1000 accessions of bean germplasm from Slovene gene bank at the Agricultural Institute of Slovenia (46°03′N, 14°31′E; 297 m above sea level), being part of the National plant gene bank. Majority of the studied bean germplasm, i.e., 936 accessions, is Slovenian origin, while the minority Slovakian (60 accessions) or Hungarian origin (4 accessions). Bean germplasm, which consists of common and runner bean collections, was evaluated using morphological seed characteristics. Several* Phaseolus* spp. seed descriptors, which have been adopted by International Union for the Protection of New Varieties of Plants (UPOV), Community Plant Variety Office (CPVO), International Board for Plant Genetic Resources (IBPGR), and Improvement of sustainable Phaseolus production in Europe for human consumption (Phaselieu), were applied to characterize a total of 953 accessions of common and 47 accessions of runner bean. Both bean germplasm collections are conserved in glass jars under medium-term storage conditions at the temperature of 4°C.

Additionally, we have also observed 24 accessions with known phaseolin type (previously determined by SDS-PAGE) as standards/anchors for the Andean/Mesoamerican/mixed germplasm within whole collection. Standards for the Andean germplasm (9) are representing the accessions with T (“Tandergreen”) phaseolin type and standards for Mesoamerican germplasm (7) are representing the accessions with S (“Sanilac”) phaseolin type. Subset group with putative hybrids between both gene pools (8) are associated with C (“Contender”) phaseolin type which originated from Andean gene pool but also represents mixed germplasm between combination of T and S phaseolin types. Table with the standard/anchor accessions are added to the supplementary data (Table S1).

### 2.2. Seed Evaluation Using Quantitative Characteristics

Quantitative seed descriptors included the evaluation of the following six characteristics: seed length (L) [mm]; seed thickness (T) [mm]; seed width (W) [mm]; seed length/width ratio (L/W); seed width/thickness ratio (W/T); and 100 (for common bean) or 10 (for runner bean) seed weight [g]. For each accession the three principal axial dimensions (L, T, W) on 10-randomly selected fully developed undamaged seeds were measured using a digital Vernier calliper (Mitutoyo 500-181-30, Japan) reading to 0.1 mm. L was measured as the highest parallel to the hilum, T as lowest parallel to the hilum, and W from hilum to the opposite side. The L/W and W/T were calculated from raw seed size data. For common bean the 100 seed weight was measured in four repetitions using an electronic seed counter (Condator, Pfeuffer GmbH, Germany) and electronic balance weighing to 0.01 g. Since there was a limited quantity of seeds available in runner bean germplasm collection, the 10 seed weight within each accession was measured in one repetition only.

### 2.3. Seed Evaluation Using Qualitative Characteristics

Qualitative seed descriptors included visual assessment of the following eight characteristics: seed colour (light, dark, colour mixture); number of seed colours (one, two, more than two); primary/main seed colour (white, green, grey, yellow, beige, brown, red, violet, black, and additionally beige/brown, beige/violet and beige/grey); predominant secondary seed colour (grey, yellow, beige, brown, red, violet, black, and additionally white, green or greenish, light/dark brown and brown/red); distribution of secondary seed colour (around hilum, on half of seed, on entire seed); seed veining (weak, medium, strong); seed shape (round/circular, oval/circular to elliptic, cuboid/elliptic, kidney shaped, truncated); seed colour (primary and secondary) and coat pattern (assessment includes a description of seed colour, shape, size and code) [19–21].

### 2.4. Statistical Analysis

The differences among the common and runner bean accessions were analysed through a general linear model procedure and least-squares mean tests (Statgraphics Centurion XVI 2009), at 0.05 level of significance. Statistics included mean, range (min, max), standard deviation (SD), coefficient of variation (CV), and analysis of variance (ANOVA). Correlations were obtained as Pearson's correlation coefficients. Principal component analysis (PCA) and cluster analysis were performed to reveal the most influential seed characteristics that discriminated among the accessions. Biplots for common and runner bean germplasms were constructed for two principal components, showing the accessions and the most influential seed characteristics. Dendrograms were conducted to combine individual variables into larger clusters using Ward's method and squared Euclidian distance.

## 3. Results and Discussion

### 3.1. Seed Characterization of Common Bean (*Phaseolus vulgaris* L.) Germplasm

A total of fourteen seed morphological characteristics, i.e., quantitative (L; T; W; L/W; W/T; and 100 seed weight) and qualitative (seed colour; number of seed colours; primary/main seed colour; predominant secondary seed colour; distribution of secondary seed colour; seed veining; seed shape; and seed colour (primary and secondary) and coat pattern), were evaluated. In the upper part of Table 1 are presented summary statistics for six quantitative common bean seed characteristics. The mean values for seed size characteristics based on all 953 accessions in common bean germplasm collection were for L 13.63 ± 1.86 mm, for T 6.70 ± 0.94 mm and for W 8.22 ± 1.08 mm (see Table 1). The minimum and maximum values ranged between 8.06 and 19.98 mm for L, 4.24 and 9.56 mm for T, and 5.06 and 12.63 mm for W. For characteristic 100 seed weight the mean value of all 953 accessions was 51.13 ± 13.84 g, while the minimum and maximum values ranged between 19.32 and 98.39 g. The highest coefficient of variation was calculated for the 100 seed weight (27.06%) and the lowest for W/T (12.64%). As discussed by Rana et al. [22] for 4274 common bean accessions conserved in Indian gene bank, L ranged 5.0–20.3 mm, W 2.0–12.0 mm and 100 seed weight 3.5–96.3 g. Similarly, Kara et al. [23] reported for 12 registered Turkish common bean genotypes L 9.1–17.8 mm, W 5.8–10.0 mm, T 4.6–6.0 mm and 100 seed weight 18.0–65.6 g. As discussed by Giurcă [24] for 9 common beans originating from northern Romania and western Ukraine, L ranged 11.8–18.0 mm, W 7.4–9.7 mm, T 4.4–6.9 mm and 100 seed weight 34.3–54.2 g. Logozzo et al. [4] evaluated 533 accessions of the European common bean germplasm and reported accessions with L 12.0–13.9 mm (35.5%), W 7.1–8.0 mm (33.0%) and T 5.0–5.9 mm (37.1%) were the most frequent.

**Figure 1:**
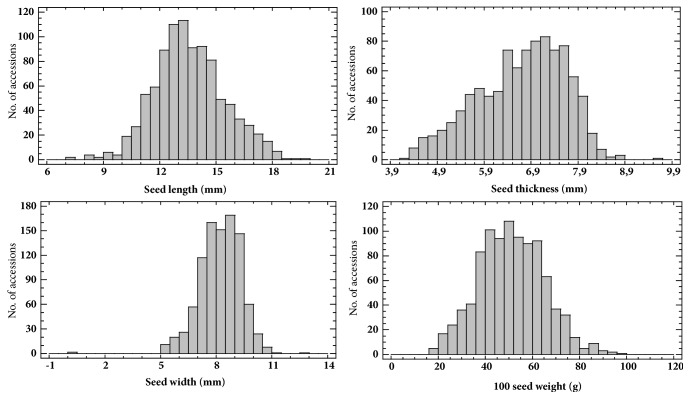
Frequency distribution of 953 common bean accessions (*Phaseolus vulgaris* L.) for quantitative seed characteristics.

**Figure 2:**
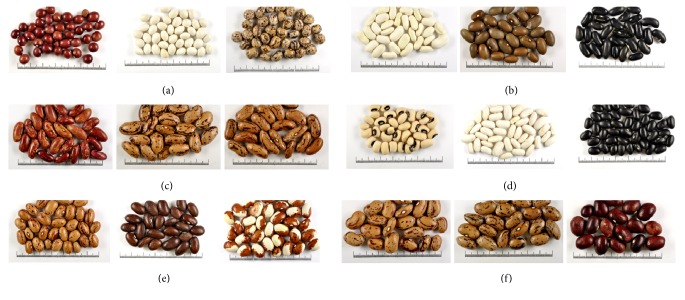
Photo examples for common bean accessions (*Phaseolus vulgaris* L.) groups distribution according to seed length: (a) small, (b) medium, and (c) large; and 100 seed weight: (d) low-weight, (e) medium-weight, and (f) high-weight.

All seed characteristics measured quantitatively or assessed qualitatively showed wide range of variation among all common bean accessions evaluated. Frequency distribution graphs of 953 common bean accessions for quantitative seed characteristics are shown in Figure 1.  Figure 2 shows photo examples for common bean accessions groups distribution according to L (a, b, c) and 100 seed weight (d, e, f). Based on quantitative measurements, the common bean accessions were classified according to the L into three groups, i.e., small, medium and large (see Figure 2). The first group included accessions with small seeds and the L < 10.0 mm (18 accessions or 2%); the second group accessions with medium seeds measuring from 10.0 to 15.0 mm (734 accessions or 77%); and the third group accessions with large seeds and L > 15.0 mm (201 accessions or 21%). Similarly, the common bean accessions were classified according to the 100 seed weight into three groups, i.e., low-weight, medium-weight and high-weight (see Figure 2). Low-weight seeds group included common bean accessions with 100 seed weight < 35.0 g (112 accessions or 12%); the medium-weight seeds group accessions with 100 seed weight measuring from 35.0 to 75.0 g (801 accessions or 84%); and the high-weight seeds group accessions with 100 seed weight > 75.0 g (40 accessions or 4%).

**Figure 3:**
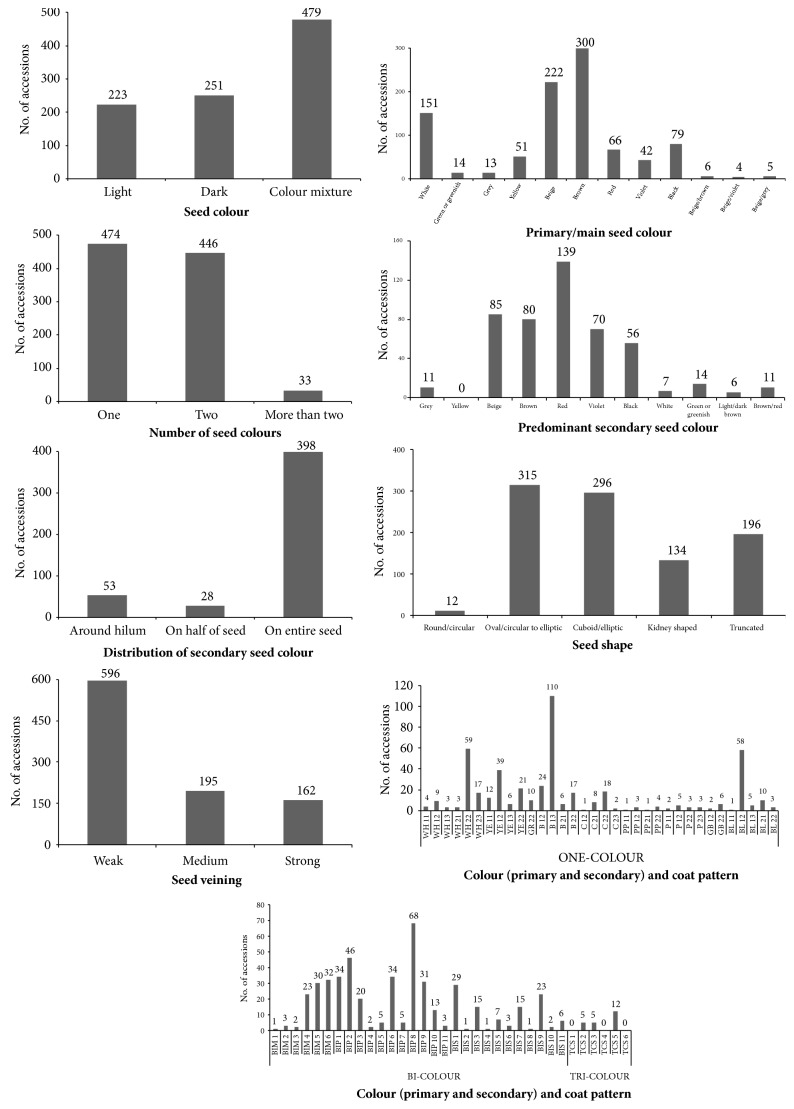
Frequency distribution of 953 common bean accessions (*Phaseolus vulgaris* L.) for qualitative seed characteristics.

Frequency distribution graphs of 953 common bean accessions for qualitative seed characteristics are shown in Figure 3. For characteristic seed colour 479 common bean accessions or 50.3% belonged to the colour mixture class, 251 accessions or 26.3% had dark-coloured seeds (brown, purple, black, etc.), and 223 accessions or 23.4% light-coloured seeds (white, pale yellow, pale brown, etc.). For characteristic number of seed colours 474 common bean accessions or 49.7% had one colour, 446 accessions or 46.8% two colours, and 33 accessions or 3.5% more than two colours per seed. The most abundant primary/main seed colours among all 953 common bean accessions were brown (300 accessions), beige (222 accessions), white (151 accessions), black (79 accessions) and red (66 accessions). The most abundant predominant secondary seed colours among 479 common bean accessions with colour mixture were red (139 accessions), beige (85 accessions), brown (80 accessions) and violet (70 accessions). Distribution of secondary seed colour was for 398 accessions on the entire seed, for 53 accessions around the hilum and for 28 accessions on the half of seed. The seed shape was oval/circular to elliptic for 315 accessions, cuboid/elliptic for 296 accessions and truncated for 196 accessions. For characteristic seed veining 596 accessions had weak, 195 accessions medium and 162 accessions strong seed veining. The characteristic seed colour (primary and secondary) and coat pattern was divided into nine classes with additional subclasses. Among one-colour class the most accessions were categorized according to Phaselieu descriptor into B 13, i.e., brown round large seeds (110 accessions), followed by WH 22, i.e., white long medium seeds (59 accessions), and BL 12, i.e., black round medium (58 accessions). A total of 455 accessions were classified into bicolour class, among which 261 were Pinto type (BIP), 103 broad striped type and 91 constant mottled type. Finally, a total of 22 accessions were classified into tricolour class. Regarding results by Logozzo et al. [4] on 533 accessions of the European common bean germplasm, the accessions with cuboid seed shape (34.9%), maroon (i.e., dark brownish red; 44.3%) and white (28.1%) seed darker colour were the most frequent. Additionally, the maroon seeds were 90.3% Andean, while the white seeds were 52% phaseolin “S” types. As discussed by Piergiovanni and Lioi [25] among 168 Italian common bean landraces according to the seed coat types, white coat seed populations and Borlotto types were the most represented.

### 3.2. Seed Characterization of Runner Bean (*Phaseolus coccineus* L.) Germplasm

A total of fourteen seed characteristics, i.e., qualitative (L; T; W; L/W; W/T; and 10 seed weight) and quantitative (same as for common bean), were evaluated. In the lower part of Table 1 are presented summary statistics for six quantitative runner bean seed characteristics. The mean values for seed size characteristics based on all 47 accessions in runner bean germplasm collection were for L 20.53 ± 2.06 mm, for T 8.70 ± 0.89 mm and for W 12.71 ± 1.17 mm (see Table 1). The minimum and maximum values ranged between 17.50 and 27.16 mm for L, 5.16 and 10.95 mm for T, and 10.03 and 16.46 mm for W. For characteristic 10 seed weight was the mean value of all 47 accessions 13.47 ± 3.45 g, while the minimum and maximum values ranged between 7.56 and 26.70 g. The highest coefficient of variation was calculated for 10 seed weight (25.62%) and the lowest for L/W (5.88%). As discussed elsewhere [6, 24] runner bean L ranged 18.6–25.0 mm, W 12.1–14.0 mm; T 7.7–12.3 mm and 10 seed weight 10.0–14.0 g.

**Figure 4:**
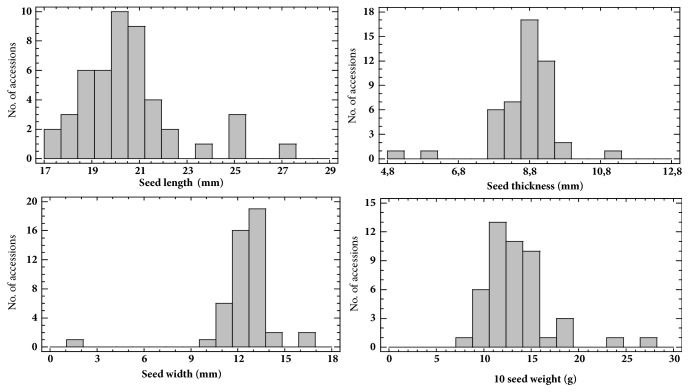
Frequency distribution of 47 runner bean accessions (*Phaseolus coccineus* L.) for quantitative seed characteristics.

**Figure 5:**
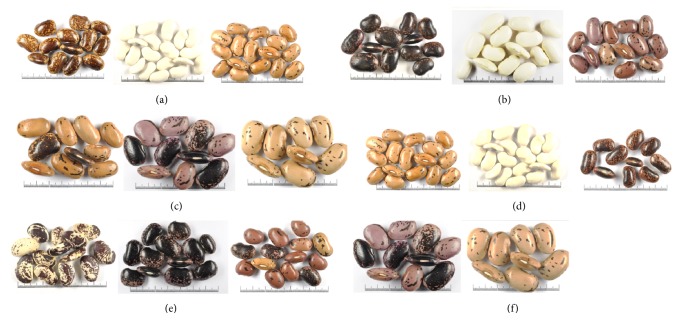
Photo examples for runner bean accessions (*Phaseolus coccineus* L.) groups distribution according to seed length: (a) small, (b) medium, and (c) large; and 10 seed weight: (d) low-weight, (e) medium-weight, and (f) high-weight.

Similarly to common bean, all seed characteristics measured quantitatively or assessed qualitatively showed wide range of variation among runner bean accessions evaluated. Frequency distribution graphs of 47 runner bean accessions for quantitative seed characteristics are shown in Figure 4.  Figure 5 shows photo examples for runner bean accessions groups distribution according to L (a, b, c) and 10 seed weight (d, e, f). Based on the quantitative measurements, the accessions of runner bean were classified according to the L into three groups, i.e., small, medium and large (see Figure 5). The first group included runner bean accessions with small seeds and the L < 20.0 mm (20 accessions or 43%); the second group accessions with medium seeds measuring from 20.0 to 25.0 mm (23 accessions or 49%); and the third group accessions with large seeds and L > 25.0 mm (4 accessions or 8%). Similarly were accessions of runner bean classified according to the 10 seed weight into three groups, i.e., low-weight, medium-weight and high-weight (see Figure 5). Low-weight seeds group included common bean accessions with 10 seed weight < 10.0 g (4 accessions or 9%); the medium-weight seeds group accessions with 10 seed weight measuring from 10.0 to 20.0 g (41 accessions or 87%); and the high-weight seeds group accessions with 10 seed weight > 20.0 g (2 accessions or 4%).

**Figure 6:**
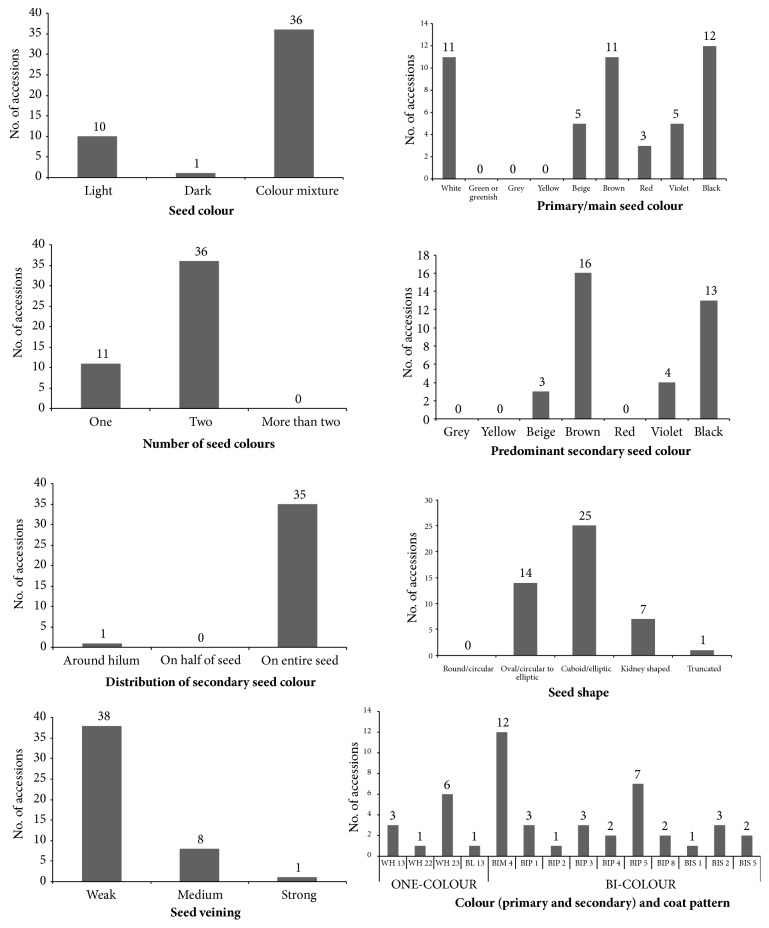
Frequency distribution of 47 runner bean accessions (*Phaseolus coccineus* L.) for qualitative seed characteristics.

Frequency distribution graphs of 47 runner bean accessions for qualitative seed characteristics are shown in Figure 6. For characteristic seed colour 36 runner bean accessions belonged to the colour mixture class, 10 accessions had light-coloured seeds and 1 accession dark-coloured seeds. For characteristic number of seed colours 11 runner bean accessions had one colour and 36 accessions two colours per seed. The most abundant primary/main seed colours among all 47 runner bean accessions were black (12 accessions), brown (11 accessions) and white (11 accessions). The most abundant predominant secondary seed colours among 36 runner bean accessions with colour mixture were brown (16 accessions) and black (13 accessions). Distribution of secondary seed colour was for 35 accessions on the entire seed and for 1 accession around the hilum. The seed shape was cuboid/elliptic for 25 runner bean accessions, oval/circular to elliptic for 14 accessions, kidney shaped for 7 accessions and truncated for 1 accession. For characteristic seed veining 38 accessions had weak, 8 accessions medium and 1 accession strong seed veining. The characteristic seed colour (primary and secondary) and coat pattern was divided into nine classes with additional subclasses. Among one-colour class the most accessions were categorized according to Phaselieu descriptor into WH 23, i.e., white long large seeds (6 accessions) and WH 13, i.e., white round large seeds (3 accessions). A total of 36 accessions were classified into bicolour class, among which 18 were Pinto type (BIP), 12 constant mottled type and 6 broad striped type. As described by Rodriguez et al. [15] within runner bean (*Phaseolus coccineus* L.) three botanical varieties exist, i.e., var.* albiflorus* with white seed colour, var.* bicolour* with beige seed and brown pattern colour, and var.* coccineus* with purple seed and black pattern colour.

### 3.3. Multivariate Analyses

The relationship between all fourteen seed characteristics, i.e., quantitative and qualitative, was evaluated for common and runner bean germplasm with the Fisher's least significant difference (LSD) method. The test was performed in order to determine which seed characteristics are significantly different from others. The results showed that characteristic seed colour (primary and secondary) and coat pattern was in statistically significant correlation with all the seed characteristics, in both bean germplasm collections.

**Figure 7:**
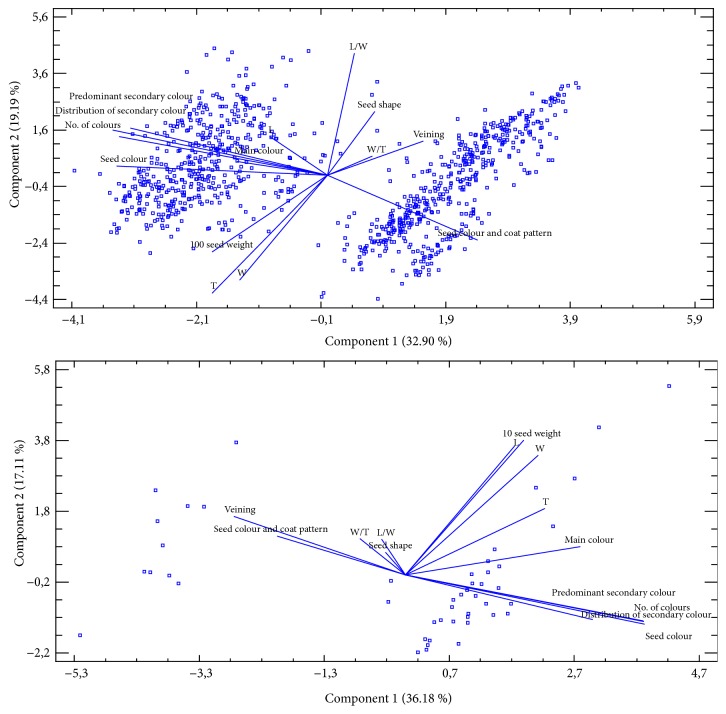
Biplot of total of fourteen seed characteristics for 953 accessions of common bean (top) and 47 accessions of runner bean germplasm (bottom). L, seed length; T, seed thickness; W, seed width; L/W, seed length/width ratio; W/T, seed width/thickness ratio.

The purpose of PCA analysis was to obtain a small number of linear combinations for fourteen variables which represent the majority of variability in the data within each bean germplasm collection. Based on an eigenvalue of greater than or equal to 1.0, 4 components were obtained for common bean germplasm (953 accessions), which together comprise 75.03% of the variability of the original data. PCA biplot in Figure 7 (top) defined Components 1 and 2, which together explained 52.09% of the total variance for fourteen variables in common bean germplasm. The Component 1 represented 32.90% and Component 2 19.19% of the total variance. The qualitative seed characteristics distribution of secondary seed colour, seed colour, number of seed colours and the secondary seed colour were the major contributors to Component 1. The quantitative seed characteristics L/W, T, and W were the major contributors to Component 2. The accessions of common bean germplasm were well separated into 3 major groups (see Figure 7).

Based on an eigenvalue of greater than or equal to 1.0, 4 components were obtained for runner bean germplasm (47 accessions), which together comprise 80.16% of the variability of the original data. PCA biplot in Figure 7 (bottom) defined Components 1 and 2, which together represent 53.29% of the total variance for fourteen variables in runner bean germplasm. The Component 1 represented 36.18% and Component 2 17.11% of the total variance. The qualitative seed characteristics number of seed colours, distribution of secondary seed colour, seed colour, secondary seed colour and seed veining were the major contributors to Component 1. The quantitative seed characteristics 10 seed weight, L, and T were the major contributors to Component 2.

**Figure 8:**
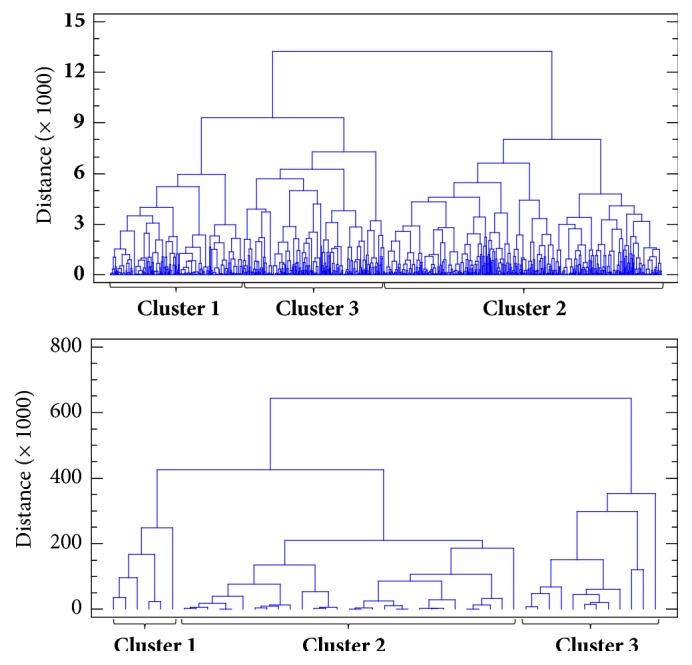
Dendrogram for 953 accessions of common bean (top) and 47 accessions of runner bean (bottom) germplasm according to fourteen seed characteristics.

Based on the cluster analyses performed on fourteen seed characteristics, all 953 common bean accessions were grouped into 3 clusters and each cluster was found to have varied number of accessions (see Figure 8 and Table 2) indicating the gene pool of origin (Mesoamerican gene pool origin, Andean gene pool origin and one subset group with putative hybrids between both gene pools). Dendrogram for accessions of common bean germplasm is presented in Figure 8 (top), where the combination of 14 individual variables established three main clusters of genotypes with standard accessions for the phaseolin type included. For this reason, it was not necessary to include pure American genotypes while we had internal standards for phaseolin type already within the collection. The highest number of accessions were ranked in cluster 2 (475), followed by cluster 3 (245) and cluster 1 (227). The quantitative seed characteristics mean values of accessions grouped into each cluster showed that accessions in cluster 1 had the lowest L (12.84 mm), L/W (1.48) and W/T (1.19), and the highest T (7.36 mm), W (8.73 mm) and 100 seed weight (55.15 g). Furthermore, accessions in cluster 2 had the highest L (13.95 mm), while accessions in cluster 3 had the lowest T (5.68 mm), W (7.39 mm) and 100 seed weight (41.06 g), and the highest L/W (1.85) and W/T (1.33). Among qualitative seed characteristics all accessions in cluster 1 had only one seed colour, which was for most accessions dark (83.70%), while most of accessions in cluster 2 had seeds with colour mixture (99.58%). The most of accessions in cluster 3 had one seed colour (99.59%), which was for most accessions (75.10%) light (Table 2). Comparing to the Indian common bean collection, 10 genetically diverse clusters were obtained regarding the phenological, morphological, and agricultural traits of common bean [21].

Similarly to the common bean, based on the cluster analyses performed on fourteen seed characteristics, all 47 runner bean accessions were grouped into 3 clusters with varied number of accessions (see Figure 8 and Table 3). Dendrogram for accessions of runner bean germplasm is presented in the Figure 8 (bottom). The highest number of accessions were ranked in cluster 2 (29), followed by cluster 3 (12) and cluster 1 (6). The quantitative seed characteristics mean values of accessions grouped into each cluster showed that accessions in cluster 1 had the highest L (24.38 mm), T (9.71 mm), W (14.66 mm), L/W (1.69) and 10 seed weight (20.08 g). Furthermore, accessions in cluster 2 had the lowest L (19.88 mm), L/W (1.60), W/T (1.39) and 10 seed weight (12.50 g), while accessions in cluster 3 had the lowest T (8.01 mm) and W (12.26 mm), and the highest W/T (1.57). Among qualitative seed characteristics all accessions in cluster 1 had seeds with colour mixture (100.00%), mainly with beige as primary/main seed colour and brown as secondary seed colour (66.67%). Accessions in cluster 2 had seeds with colour mixture (100.00%), mainly with black or brown as primary/main and secondary seed colour. The most of accessions in cluster 3 had one seed colour (91.67%) which was for most accessions (83.33%) light (Table 3). As discussed by Schwember et al. [6] extensive characterizations of runner bean germplasm are great challenges and opportunities in the future that would increase its cultivation on a broader scale worldwide.

## 4. Conclusion

This manuscript describes for the first time large-scale morphological seed characterization of the common and runner bean collection germplasm conserved in the Slovenian gene bank. The germplasm evaluated has a wide range of morphological variability based on fourteen seed characteristics. The study encompassed 953 accessions of common and 47 accessions of runner bean. Seeds of each accession were evaluated for quantitative characteristics: L (range 7.3–27.2 mm); T (range 4.2–11.0 mm); W (range 0.3–16.5 mm); L/W (range 0.4–2.6 mm); W/T (range 0.6–2.2 mm); and 100 for common (range 19.3–98.4 mm) or 10 for runner (range 7.6–26.7 mm) bean seed weight. Furthermore, seeds were evaluated using qualitative characteristics: seed colour (50.3% of common beans and 76.6% of runner beans were colour mixture), number of seed colours (49.7% of common beans had one colour and 76.6% of runner beans two colours per seeds), primary/main seed colour (31.5% of common beans were brown and 25.5% of runner beans black), predominant secondary seed colour (14.6% of common beans had red colour and 34.0% of runner beans brown colour), distribution of secondary seed colour (41.8% of common beans and 74.5% of runner beans had secondary colour distributed on the entire seed), seed veining (62.5% of common beans and 80.9% of runner beans had weak seed veining), seed shape (33.1% of common beans had oval/circular to elliptic and 53.2% of runner beans cuboid/elliptic seed shape), and seed colour and coat pattern (11.5% of common beans had large and one-colour brown seeds; 25.5% of runner beans had bicolour constant mottled seeds). The results of seed characterization indicate the origin (Andean, Mesoamerican, putative hybrids between gene pools) and domestication pathways of common and runner beans. To date, this is the first study reporting the morphological characteristics and comparisons of whole common and runner bean germplasm conserved in one of the Central European collections. The results obtained in this study are serving as the useful information on genetic diversity of common and runner bean accessions at the Slovene gene bank, which could be used for development of new bean varieties for studied seed characteristics. Nondestructive screening test based on the seed characterization of large bean germplasm is shown to be an informative, noninvasive, and suitable tool for distinction of bean accessions according to the gene pool origin.

## Figures and Tables

**Table 1:** Summary statistics for six quantitative seed characteristics in 953 accessions of common (*Phaseolus vulgaris* L.) and 47 accessions of runner bean (*Phaseolus coccineus* L.).

Characteristics	Range	Mean ± SD	CV (%)
*Common bean*			
L (mm)	8.06 – 19.98	13.63 ± 1.86	13.63
T (mm)	4.24 – 9.56	6.70 ± 0.94	13.97
W (mm)	5.06 – 12.63	8.22 ± 1.08	13.09
L/W	1.08 – 2.64	1.69 ± 0.28	16.57
W/T	0.97 – 2.49	1.24 ± 0.16	12.64
100 seed weight (g)	19.32 – 98.39	51.13 ± 13.84	27.06

*Runner bean*			
L (mm)	17.50 – 27.16	20.53 ± 2.06	10.05
T (mm)	5.16 – 10.95	8.70 ± 0.89	10.25
W (mm)	10.03 – 16.46	12.71 ± 1.17	9.20
L/W	1.42 – 1.89	1.62 ± 0.10	5.88
W/T	1.27 – 2.17	1.48 ± 0.16	10.79
10 seed weight (g)	7.56 – 26.70	13.47 ± 3.45	25.62

SD, standard deviation; CV, coefficient of variation; L, seed length; T, seed thickness; W, seed width; L/W, seed length/width ratio; W/T, seed width/thickness ratio.

**Table 2:** Means or descriptive characters of different variables in 3 clusters for 953 accessions of common bean (*Phaseolus vulgaris* L.).

Cluster variables	Cluster 1	Cluster 2	Cluster 3
*Quantitative seed characteristic *			
L (mm)	12.84	13.95	13.65
T (mm)	7.36	6.91	5.68
W (mm)	8.73	8.37	7.39
L/W	1.48	1.69	1.85
W/T	1.19	1.22	1.33
100 seed weight (g)	55.15	54.41	41.06

*Qualitative seed characteristic*			
Seed colour	Dark colour (83.70^*∗*^)	Colour mixture (99.58^*∗*^)	Light colour (75.10^*∗*^)
Number of seed colours	One (100.00^*∗*^)	Two (93.05^*∗*^)	One (99.59^*∗*^)
Primary/main seed colour	Brown (51.98^*∗*^)	Brown (28.63^*∗*^)	White (36.33^*∗*^)
Predominant secondary seed colour	None (99.56^*∗*^)	Red (29.05^*∗*^)	None 99.59^*∗*^)
Distribution of secondary seed colour	Without secondary colour (100.00^*∗*^)	On entire seed (82.95^*∗*^)	Without secondary colour (99.59^*∗*^)
Seed veining	Weak (75.33^*∗*^)	Weak (73.68^*∗*^)	Strong (40.82^*∗*^)
Seed shape	Oval/circular to elliptic (55.07^*∗*^)	Oval/circular to elliptic (36.63^*∗*^)	Cuboid/elliptic (39.59^*∗*^)
Seed colour and coat pattern	One-colour: Brown (48.90^*∗*^)	Bi-colour: Pinto T type (54.53^*∗*^)	One-colour: White (35.92^*∗*^)

Number of accessions	227	475	245

*∗*, % of main characteristic within each cluster; L, seed length; T, seed thickness; W, seed width; L/W, length/width ratio; W/T, width/thickness ratio.

**Table 3:** Means or descriptive characters of different variables in 3 clusters for 47 accessions of runner bean (*Phaseolus coccineus* L.).

Cluster variables	Cluster 1	Cluster 2	Cluster 3
*Quantitative seed characteristic *			
L (mm)	24.38	19.88	20.17
T (mm)	9.71	8.78	8.01
W (mm)	14.66	12.49	12.26
L/W	1.69	1.60	1.66
W/T	1.51	1.39	1.57
10 seed weight (g)	20.08	12.50	12.54

*Qualitative seed characteristic*			
Seed colour	Colour mixture (100.00^*∗*^)	Colour mixture (100.00^*∗*^)	Light colour (83.33^*∗*^)
Number of seed colours	Two (100.00^*∗*^)	Two (100.00^*∗*^)	One (91.67^*∗*^)
Primary/main seed colour	Beige (66.67^*∗*^)	Black or brown (37.93^*∗*^)	White (91.67^*∗*^)
Predominant secondary seed colour	Brown (66.67^*∗*^)	Black or brown (37.93^*∗*^)	None (91.67^*∗*^)
Distribution of secondary seed colour	On entire seed (100.00^*∗*^)	On entire seed (96.55^*∗*^)	None (91.67^*∗*^)
Seed veining	Weak (83.33^*∗*^)	Weak (100.00^*∗*^)	Medium (58.33^*∗*^)
Seed shape	Cuboid/elliptic (83.33^*∗*^)	Cuboid/elliptic (62.07^*∗*^)	Oval/circular to elliptic (41.67^*∗*^)
Seed colour and coat pattern	Bi-colour: Pinto T type (50.00^*∗*^)	Bi-colour: Pinto T type (48.28^*∗*^)	One-colour: White (83.33^*∗*^)

Number of accessions	6	29	12

*∗*, % of main characteristic within each cluster; L, seed length; T, seed thickness; W, seed width; L/W, length/width ratio; W/T, width/thickness ratio.

## Data Availability

The data used to support the findings of this study are included within the article.

## Abbreviations

AFLP: Amplified fragment length polymorphism
cpSSRs: Chloroplast microsatellites or simple sequence repeats
L: Seed length
T: Seed thickness
W: Seed width
L/W: Length/width ratio
W/T: Width/thickness ratio
PCA: Principal component analysis.

## Supplementary Materials

Supplementary Materials Table  S1: List of standard common bean (*Phaseolus vulgaris* L.) accessions with known phaseolin type.
