# Supplementary material for: Morphological Seed Characterization of Common (Phaseolus vulgaris L.) and Runner (Phaseolus coccineus L.) Bean Germplasm: A Slovenian Gene Bank Example
Source: Biomed Res Int. 2019 Jan 16;2019:6376948. doi: 10.1155/2019/6376948 (PMC6354170; doi:10.1155/2019/6376948)
Supplement: Supplementary Materials — Table S1: List of standard common bean (Phaseolus vulgaris L.) accessions with known phaseolin type. [file 6376948.f1.docx]

Supplementary Table

Table S1: List of standard accessions with known phaseolin type

| **Internal Acession number** | **Source (GB)** | **Fazeolin type (SDS-PAGE)** | **Country of origin** |
| --- | --- | --- | --- |
| PHA914 | AIS | C | Slovenia |
| PHA390 | AIS | C | Slovenia |
| PHA315 | AIS | C | Slovenia |
| PHA627 | AIS | C | Slovenia |
| PHA222 | AIS | C | Slovenia |
| PHA29 | AIS | C | Slovenia |
| PHA181 | AIS | C | Slovenia |
| PHA59 | AIS | C | Slovenia |
| PHA371 | AIS | S | Slovenia |
| PHA368 | AIS | S | Slovenia |
| PHA7 | AIS | S | Slovenia |
| PHA915 | AIS | S | Slovenia |
| PHA97 | AIS | S | Slovenia |
| PHA55 | AIS | S | Slovenia |
| PHA245 | AIS | S | Slovenia |
| PHA90 | AIS | T | Slovenia |
| PHA306 | AIS | T | Slovenia |
| PHA131 | AIS | T | Slovenia |
| PHA498 | AIS | T | Slovenia |
| PHA336 | AIS | T | Slovenia |
| PHA388 | AIS | T | Slovenia |
| PHA318 | AIS | T | Slovenia |
| PHA309 | AIS | T | Slovenia |
| PHA418 | AIS | T | Slovenia |

AIS, Agricultural institute of Slovenia.
